# Retraction: Facile fabrication of water-dispersible AgInS_2_ quantum dots and mesoporous AgInS_2_ nanospheres with visible photoluminescence

**DOI:** 10.1039/d0ra90105a

**Published:** 2020-10-19

**Authors:** Laura Fisher

**Affiliations:** Royal Society of Chemistry, Thomas Graham House, Science Park Milton Road Cambridge CB4 0WF UK advances-rsc@rsc.org

## Abstract

Retraction of ‘Facile fabrication of water-dispersible AgInS_2_ quantum dots and mesoporous AgInS_2_ nanospheres with visible photoluminescence’ by Hui Jin *et al.*, *RSC Adv.*, 2015, **5**, 68287–68292. DOI: 10.1039/C5RA11545K

(1) The Royal Society of Chemistry has been notified by the Office of Academic Research, Qingdao University that the authorship, affiliations and acknowledgements of this paper are incorrect. They informed us that “Rijun Gui has confirmed that he independently completed the experimental research before he joined Qingdao University. Without their knowledge and prior notice, he signed the names of irrelevant researchers (Hui Jin, Zonghua Wang, Jianfei Xia, Min Yang, Feifei Zhang and Sai Bi) in his paper and added the Funding numbers of the irrelevant researchers in his papers without authorization. Rijun Gui confirmed that these irrelevant researchers did not participate in experimental researches reported in his paper and they did not provide financial support. They have no contribution to the paper.” They concluded that “the names of the irrelevant researchers without contribution are required to be deleted from the authorship of the paper, and their funding numbers without providing financial support are required to be deleted from the “Acknowledgements”.” The corrected authorship list and affiliations for this paper are as follows:

Rijun Gui*^a^

^a^School of Chemistry and Chemical Engineering, Shanghai Jiao Tong University, Shanghai 200240, P. R. China, email: guirijun@163.com

The corrected Acknowledgements for this paper are as follows:

This work was financially supported by the Postdoctoral Science Foundation of Shanghai (13R21413800).

(2) The Royal Society of Chemistry hereby wholly retracts this *RSC Advances* article due to concerns with the reliability of the data in the published article.

The TEM image in Fig. 2a contains duplications of the same particles within the image, which indicates that it has been manipulated.

The TEM image in Fig. 2c duplicates data from another publication by Tan *et al.*, but representing different materials.^1^

The TEM image in Fig. 2d duplicates data from another publication by Tan *et al.*, but representing different materials.^2^

Given the number and significance of the concerns about the validity of the data, the findings presented in this paper are no longer reliable.

Rijun Gui requested to retract this article due to the incorrect authorship, but opposes the wording in this retraction notice.

Signed: Laura Fisher, Executive Editor, *RSC Advances*

Date: 21^st^ September 2020

## Supplementary Material
